# Pachydermodactyly: A Diagnostic Pitfall in Adolescents Referred to Pediatric Rheumatology for Suspected Juvenile Idiopathic Arthritis

**DOI:** 10.3390/children13060748

**Published:** 2026-05-27

**Authors:** Andrei-Ioan Munteanu, Delia-Maria Nicoară, Iulius Jugănaru, Raluca Asproniu, Otilia Mărginean

**Affiliations:** 1 Department XI Pediatrics, Discipline I Pediatrics, ‘Victor Babeș’ University of Medicine and Pharmacy of Timisoara, 300041 Timisoara, Romania; andrei-ioan.munteanu@umft.ro (A.-I.M.); nicoara.delia@umft.ro (D.-M.N.); raluca.asproniu@umft.ro (R.A.); marginean.otilia@umft.ro (O.M.); 2 1st Department of Pediatrics, Children’s Emergency Hospital ‘Louis Țurcanu’, 300011 Timisoara, Romania; 3 Research Center for Disturbances of Growth and Development in Children BELIVE, ‘Victor Babeș’ University of Medicine and Pharmacy of Timisoara, 300041 Timisoara, Romania; 4 Ph.D. School Department, ‘Victor Babeș’ University of Medicine and Pharmacy of Timisoara, 300041 Timisoara, Romania

**Keywords:** pachydermodactyly, pediatrics, painless swelling, juvenile idiopathic arthritis, digital fibromatosis, diagnosis of exclusion

## Abstract

**Highlights:**

**What are the main findings?**

- Pachydermodactyly was diagnosed in two male adolescents initially suspected of having juvenile idiopathic arthritis, based on systematic exclusion of inflammatory and other structural conditions through clinical, laboratory, and imaging assessment.
- Comprehensive evaluation—including ultrasound, radiography, and, in Case 2, MRI to characterize atypical bilateral soft tissue changes with perilesional hypopigmentation—showed no signs of joint inflammation, synovitis, or bone changes, confirming the non-inflammatory nature of the soft tissue swelling in both patients.

**What are the implications of the main findings?**

- Recognizing pachydermodactyly as a distinct, non-inflammatory condition is essential to avoid exposing young patients to unnecessary immunosuppressive treatments.
- Repetitive mechanical microtrauma to the fingers is one of several possible contributing factors—alongside idiopathic predisposition, genetic susceptibility, and behavioral causes—in triggering the fibrotic changes seen in this condition; addressing these behaviors through counseling may help limit disease progression.
- Close collaboration between rheumatology and dermatology is needed to correctly identify this rare condition and ensure appropriate, multidisciplinary management.

**Abstract:**

Pachydermodactyly (PDD) is a benign, non-inflammatory, non-erosive digital fibromatosis characterized by progressive, asymptomatic, periarticular soft tissue thickening predominantly affecting the proximal interphalangeal (PIP) joints. Additional localizations, including the palm and the distal interphalangeal (DIP) or metacarpophalangeal (MCP) joints, have also been reported. The etiology of PDD is multifactorial, encompassing idiopathic, trauma-induced, genetic, and behavioral factors. *Objective*: The aim of this report is to describe the clinical, imaging, and laboratory features of pachydermodactyly in two male adolescents initially referred to a pediatric rheumatology service for suspected juvenile idiopathic arthritis (JIA), highlighting the diagnostic pitfalls and differentiation criteria from inflammatory arthritis. In addition, a narrative review of cases published from 1975 to 2025 is presented to contextualize our findings within the broader literature. *Results*: Two male adolescents (aged 13 years and 5 months and 16 years) presented with progressive, painless periarticular soft tissue swelling of the PIP joints, initially raising suspicion for JIA. Comprehensive evaluation identified characteristic features of PDD in both patients, with complete absence of inflammatory markers, synovitis, or osseous changes. Case 1 was classified as mono-PDD and Case 2 as classic, trauma-associated PDD with atypical perilesional hypopigmentation, requiring MRI for definitive exclusion of infiltrative pathology. A narrative review of 15 representative published cases from 2014 to 2025 is presented, demonstrating persistent underdiagnosis and consistent misclassification as JIA across multiple clinical settings and geographic regions. *Conclusions*: PDD should be considered in the differential diagnosis of any adolescent presenting with painless digital swelling. Its recognition as a benign, non-inflammatory entity is essential to prevent unnecessary diagnostic procedures and immunosuppressive therapy. Clinical awareness and multidisciplinary assessment remain the cornerstones of accurate diagnosis and appropriate management.

## 1. Introduction

In rheumatological practice, joint swelling is a cardinal sign of inflammatory disease, reflecting periarticular edema, synovitis, and synovial fluid accumulation. In JIA, the presence of swollen and tender joints associated with restricted mobility defines disease activity and justifies rheumatological referral. However, certain rare, non-inflammatory conditions can cause periarticular thickening that mimics inflammatory joint swelling. Pachydermodactyly (PDD) is one such benign entity, characterized by progressive thickening of the periarticular skin, predominantly at the PIP joints, and is frequently confused with JIA, exposing patients to the risk of unwarranted immunosuppressive treatments [1,2].

The disorder is characterized by asymptomatic, symmetric, progressive soft tissue swelling of the PIP joints of fingers II–IV, and rarely finger V. It was first described by Basex et al. in 1973 [3] and named by Verbov in 1975, from the Greek: pachy (thick), dermos (skin), and dactylos (finger). To date, approximately 150 cases of PDD have been reported in the literature [4]; however, this figure likely underestimates the true incidence of this underdiagnosed condition.

Based on data available up to 2022, it is mostly reported in adolescent males. There are 139 patients with pachydermodactyly reported to date [5], yielding a male-to-female ratio of approximately 4:1; this ratio may have evolved as additional cases have been described in recent years. In most cases, periarticular thickening begins at puberty—around the age of 14 years—progresses insidiously over several years, and stabilizes during adolescence [6]. To reduce diagnostic confusion and delay in recognizing this entity, Chen et al. proposed, in 2015, a set of six clinical and paraclinical criteria for the diagnosis of pachydermodactyly. These include the absence of subjective symptoms and morning stiffness, lack of tenderness on palpation, typical localization of swelling on the ulnar or radial aspects of the fingers, normal inflammatory laboratory parameters, and the presence of soft tissue thickening on plain radiography. The application of these criteria allows for differentiation from inflammatory arthritides, particularly juvenile idiopathic arthritis [7].

However, it must be stressed that, in clinical practice, pachydermodactyly is fundamentally a diagnosis of exclusion. As demonstrated by Zabotti et al. [8], the diagnosis should be based on a careful clinical evaluation supported by non-invasive investigations—blood tests, radiographs, and especially ultrasonography—rather than on the fulfillment of a fixed set of criteria. Ultrasonography is particularly valuable, as it can demonstrate periarticular soft tissue thickening without synovitis, joint effusion, or increased Doppler signal, thereby excluding inflammatory arthropathy in a non-invasive, cost-effective manner [8].

When clinical features are atypical or inflammatory disease cannot be confidently excluded on clinical grounds alone, additional diagnostic procedures—including ultrasound, radiography, and, in selected cases, MRI—may be warranted to complete the exclusion workup. Importantly, establishing the correct diagnosis avoids unnecessary diagnostic procedures and unjustified immunosuppressive therapy. The etiology of PDD remains incompletely understood; while repetitive mechanical microtrauma is considered the predominant factor in most cases, idiopathic forms, genetically predisposed individuals, and associations with systemic conditions have all been reported [9].

Nonetheless, PDD may also arise idiopathically, without any identifiable triggering behavior, or in the context of inherited connective tissue disorders and other systemic conditions—indicating a multifactorial pathogenesis. Associations have been reported with conditions characterized by tissue fragility or repetitive behaviors, including Ehlers–Danlos syndrome, tuberous sclerosis, and autism spectrum disorders, including Asperger syndrome. A familial pattern has also been described, with changes detected in first-degree relatives (fathers, grandfathers) of affected individuals; family members should therefore be examined when PDD is suspected. Psychological factors, particularly anxiety and emotional stress, may promote compulsive or tic-like behaviors, contributing to the perpetuation of periarticular lesions [10]. In our two cases, the parents who accompanied the patients at the time of admission (mother and father in both cases) were clinically examined and showed no signs of periarticular soft tissue thickening, making a familial form unlikely.

## 2. Case Presentations

Two male adolescents were evaluated in the Pediatric Rheumatology Department of the Children’s Emergency Clinical Hospital “Louis Țurcanu” Timișoara, Romania, between September and October 2025. Both had been referred with a clinical suspicion of juvenile idiopathic arthritis. In each case, the same systematic approach was followed: detailed clinical history, thorough physical examination, laboratory investigations, and imaging assessment, with the extent of imaging tailored to the complexity of the presentation.

### 2.1. Case 1

#### 2.1.1. Clinical History

A 13-year-old and 5-month-old male presented with a two-year history of progressive, painless soft tissue swelling of the PIP joints of both hands. He reported no morning stiffness, no pain at rest or on exertion, and no limitation of hand function. There was no history of trauma, recent infection, psoriasis, or gastrointestinal symptoms. He denied any repetitive finger manipulation habits or compulsive behaviors. Both parents, present at admission, showed no periarticular soft tissue thickening on clinical examination, making a familial form of PDD unlikely.

#### 2.1.2. Physical Examination

**Figure 1:**
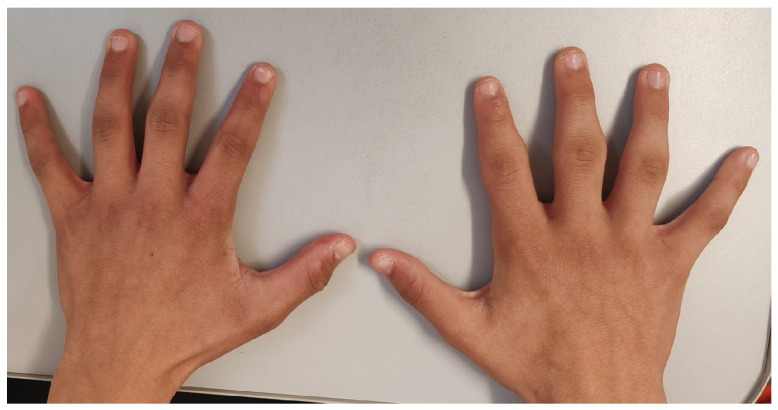
Clinical appearance of Case 1—fusiform, symmetric periarticular swelling of the PIP joints of fingers II–IV, right hand.

Examination of the hands revealed fusiform, symmetric periarticular soft tissue thickening of the PIP joints, most pronounced at fingers II–IV of the right hand (Figure 1). There was no erythema, local warmth, or tenderness on palpation. Joint mobility was fully preserved, and no fixed deformities were present. Examination of other joints and organ systems was unremarkable.

#### 2.1.3. Laboratory Investigations

Complete blood count, ESR, CRP, ASLO, rheumatoid factor, ANA, and metabolic panel were all within normal limits (Table 1). No inflammatory or autoimmune markers were elevated.

#### 2.1.4. Imaging

Bilateral soft tissue ultrasound revealed no synovial hypertrophy and no intra-articular fluid collections at any PIP joint. Periarticular soft tissue thickening involving the collateral ligaments and subcutaneous tissues was identified, most prominent at PIP II–IV of the right hand. No MRI was performed—the clinical picture and ultrasound findings were sufficient for diagnosis, consistent with the approach advocated by Zabotti et al. for typical presentations [8].

#### 2.1.5. Diagnosis

A diagnosis of PDD was established through systematic exclusion of inflammatory and structural conditions. The case was classified as idiopathic mono-PDD—no behavioral triggers identified, predominantly unilateral involvement of limited fingers—in accordance with the Sharma et al. classification [11] and Bardazzi et al. subtype criteria [12].

### 2.2. Case 2

#### 2.2.1. Clinical History

A 16-year-old male was referred for suspected JIA. He reported a four-month history of progressive, painless bilateral swelling of the PIP II–IV joints, without morning stiffness, pain, skin rash, or functional limitation. No history of trauma, recent infection, psoriasis, or gastrointestinal complaints. No neuropsychiatric history. Upon direct questioning, the patient reported habitual finger-twisting and interlocking behaviors performed unconsciously during periods of stress—consistent with repetitive mechanical microtrauma to the periarticular soft tissues. Both parents, present at admission, showed no periarticular soft tissue changes on examination, making a familial form of PDD unlikely.

#### 2.2.2. Physical Examination

**Figure 2:**
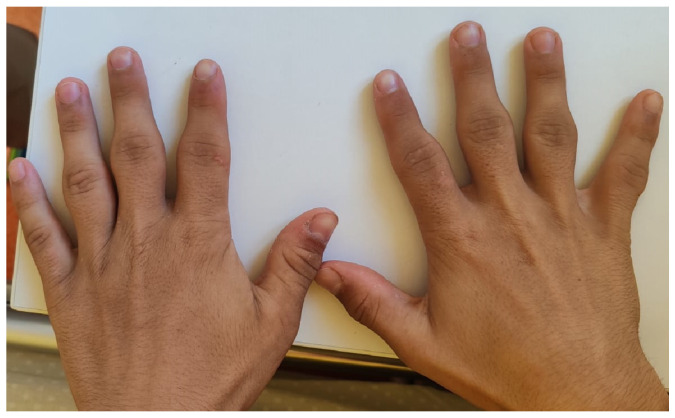
Clinical appearance of Case 2—bilateral PIP joint swelling with cutaneous thickening and perilesional hypopigmentation.

Examination revealed bilateral soft tissue enlargement on the lateral aspects of the PIP joints, predominantly affecting PIP II–IV and mildly PIP V of the right hand. The swelling was associated with cutaneous thickening and perilesional hypopigmentation (Figure 2)—a finding rarely described in PDD that raised concern for an infiltrative or pigmentation-related disorder, prompting extension of the imaging workup. No fixed joint deformities were present. Range of motion was fully preserved. There was no erythema, local warmth, or tenderness on palpation. Examination of other joints and organ systems was unremarkable.

#### 2.2.3. Laboratory Investigations

Complete blood count, ESR, ASLO, CRP, hepatic and renal function tests, rheumatoid factor, and ANA were all within normal limits (Table 1). No inflammatory or autoimmune markers were elevated.

#### 2.2.4. Imaging

**Figure 3:**
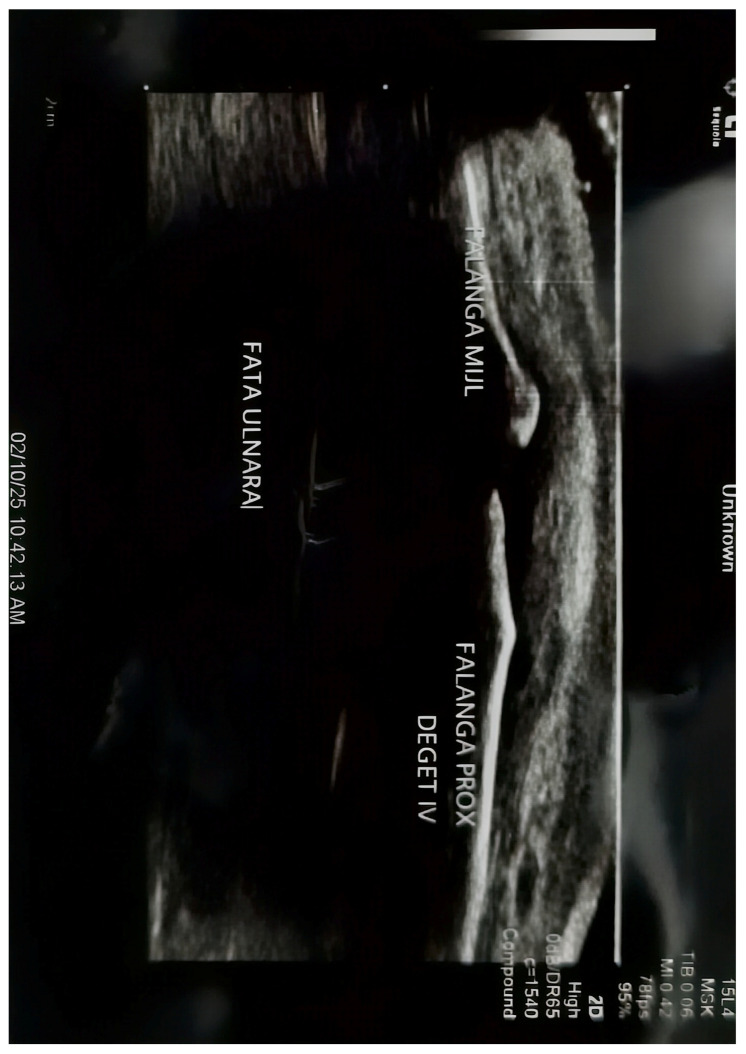
Ultrasound—periarticular soft tissue swelling on the ulnar and radial margins of the PIP joints.

**Figure 4:**
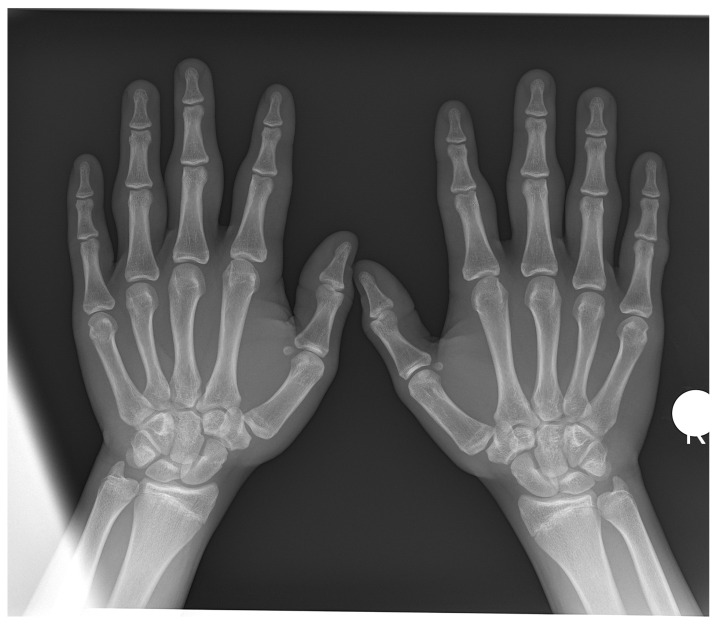
Plain radiograph of both hands (Case 2)—normal bone and joint structure, with no osseous erosions, articular space narrowing, or periosteal changes.

Ultrasound of both hands showed no synovial effusion or synovial hypertrophy at any PIP joint; periarticular soft tissue swelling was identified on the ulnar and radial margins of the affected joints (Figure 3). Plain radiography of the hands showed no osseous or articular changes (Figure 4).

**Figure 5:**
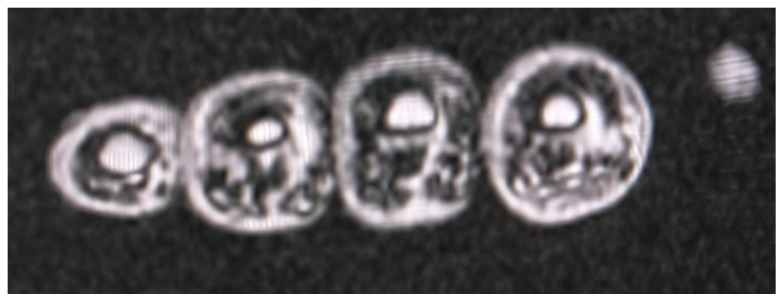
MRI of the hand—hypointense cutaneous thickening on T1, no synovitis or joint effusion.

**Figure 6:**
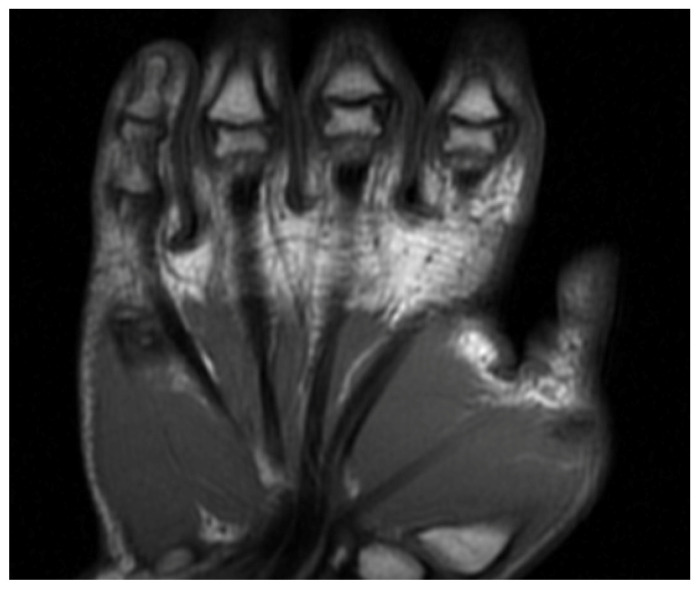
MRI of the hand—mild T2 hyperintensity of periarticular soft tissues; no tendinitis.

Given the bilateral swelling distribution, the perilesional hypopigmentation, and the need to definitively exclude infiltrative or inflammatory soft tissue pathology not fully characterizable by ultrasound alone, MRI of the hand was performed. MRI demonstrated T1 hypointense cutaneous thickening and mild T2 periarticular hyperintensity, with no joint effusion, synovitis, or tendinitis (Figure 5 and Figure 6). This pattern—fibrotic periarticular thickening without articular or bone marrow involvement—is consistent with PDD as described by Anandacoomarasamy et al. [13].

#### 2.2.5. Diagnosis

Inflammatory rheumatological pathology was excluded based on normal laboratory parameters, absence of synovitis on ultrasound, and the characteristic MRI pattern. A diagnosis of PDD was established through exclusion. The case was classified as classic trauma-induced PDD per the Sharma et al. classification [11], given bilateral multi-finger involvement and the documented repetitive behavioral trigger. The perilesional hypopigmentation represents an atypical feature not described in any other case in our narrative review.

## 3. Discussion

Despite growing awareness, PDD continues to be misdiagnosed as JIA even in tertiary pediatric rheumatology centers across Europe, as recently confirmed by Rukavina et al. in a case series of seven patients from Croatia [14]. The first systematic review of PDD, published by Vázquez Fernández et al. in 2021 and encompassing 97 studies, identified 139 patients worldwide [15]; subsequent case reports have continued to expand this number, and underdiagnosis remains likely given the benign, asymptomatic course [5]. To our knowledge, the present report represents the first description of PDD from a pediatric rheumatology service in Romania. Notably, Table 2 reveals that the clinical setting at referral is almost invariably rheumatology rather than dermatology, reflecting the symptom-driven pathway: visible “joint swelling” directs patients to rheumatologists, while the dermal fibrotic nature of the condition places it at the interface of the two specialties. Both our cases followed this same pattern, confirming that increased awareness within pediatric rheumatology—rather than dermatology alone—is the primary unmet need.

Establishing a diagnosis of pachydermodactyly typically involves a process of exclusion, particularly in the absence of other suggestive systemic signs. Due to the rarity of the condition and clinicians’ limited familiarity with it, PDD is frequently misidentified as an inflammatory arthropathy, most commonly JIA [14]. As emphasized by Zabotti et al., this exclusion process should be supported primarily by non-invasive investigations—blood tests, radiographs, and especially ultrasonography—which together allow the diagnosis to be established without the need for biopsy or MRI in typical cases.

The importance of correct diagnostic framing is further highlighted by Barnes et al., who described the histopathologic pitfalls that may lead to erroneous interpretation of biopsy specimens, emphasizing that clinical context is essential for accurate diagnosis [22].

Case 1 was classified as mono-PDD based on the unilateral, single-finger predominance of swelling. Although the classic form of PDD, as described by Bardazzi et al. (1998) [12], typically affects multiple fingers bilaterally, a localized or mono-PDD subtype has since been recognized and is described in the classification system of Sharma et al. [11] as an established variant—idiopathic or trauma-induced—characterized by involvement limited to one or a small number of fingers, sometimes unilaterally. Case 2 was classified as classic PDD induced by repetitive traumatic stress, according to the following classification [11]:

1. Classic PDD (idiopathic or trauma-induced), described mainly in male patients with multiple affected fingers;
2. Localized or mono-PDD (idiopathic or trauma-induced);
3. PDD transgrediens, when cutaneous changes extend to the palms or MCP joints;
4. Familial PDD (classic or transgrediens);
5. PDD associated with tuberous sclerosis, which may be painful [11].

The clinical features of our patients are consistent with those previously reported in the literature, supporting the non-inflammatory nature of pachydermodactyly. As shown in Table 1, the laboratory parameters of both patients were comparable and uniformly within normal limits.

The diagnosis of PDD is primarily clinical and represents a diagnosis of exclusion. Skin biopsy is not mandatory in routine practice, and its absence does not preclude diagnosis when clinical, laboratory, and imaging criteria are met. When a biopsy is performed—for example, to exclude other fibroproliferative or infiltrative conditions—histopathological examination may reveal characteristic but non-specific dermal changes, including dermal thickening through collagen accumulation (predominantly type I [23], III, and V [24]), variable hyperkeratosis and acanthosis, mild fibroblastic proliferation [25], and occasional mucin deposits [26,27]. These findings are supportive but not pathognomonic and must be interpreted within the clinical context.

Although MRI is not included among the diagnostic criteria for PDD and is not required for routine diagnosis—as illustrated by Case 1, where clinical and ultrasound findings were sufficient—its use may be justified in specific clinical scenarios characterized by diagnostic uncertainty. Indications for MRI in the workup of suspected PDD include bilateral or atypical distribution of soft tissue swelling; presence of associated cutaneous changes such as hypopigmentation or hyperpigmentation, which may raise concern for infiltrative or granulomatous disorders; inability to confidently exclude early synovitis, tenosynovitis, or deep tissue edema on ultrasound alone; atypical patient profile (e.g., absence of known behavioral triggers, younger age, female sex); and cases where a definitive non-inflammatory characterization is needed to avoid initiating immunosuppressive therapy. The MRI pattern of PDD—hypointense T1 cutaneous thickening with mild T2 periarticular hyperintensity, without synovitis, joint effusion, or bone marrow edema—was first formally described by Anandacoomarasamy et al. [13] and has since been reproduced in multiple independent reports, providing a reliable imaging fingerprint that can exclude inflammatory and infiltrative pathology in diagnostically ambiguous cases.

In Case 2, the bilateral distribution and perilesional hypopigmentation prompted MRI, which demonstrated hypointense T1 cutaneous thickening with mild T2 periarticular signal without synovitis, effusion, or tendinitis—a pattern consistent with fibrotic soft tissue changes and incompatible with active inflammatory disease. From a cost–benefit perspective, MRI should not be used indiscriminately; however, in diagnostically ambiguous cases, the cost of an unnecessary MRI is substantially lower than the cost—financial, physical, and psychological—of an erroneous immunosuppressive treatment course in an adolescent patient. Ultrasound with advanced modalities such as superb microvascular imaging and elastography may serve as a cost-effective alternative in centers where these techniques are available. In centers where advanced ultrasound modalities are available, superb microvascular imaging and elastography have been shown to characterize the fibrotic nature of PDD tissue with high specificity, offering a non-invasive, radiation-free alternative to MRI in selected cases [19].

Studies on human skin have demonstrated that exposure to repetitive mechanical stress activates cellular mechanotransduction pathways, leading to increased cell proliferation and overexpression of fibrogenesis mediators, including integrin β1, p130Cas, TGF-β1, and type I collagen. These mechanisms support the hypothesis that repetitive microtrauma (as observed in Case 2) plays a role in the development of the characteristic periarticular fibrotic thickening of pachydermodactyly [12,28]. Importantly, as demonstrated across the cases summarized in Table 2 and in the series by Rukavina et al. [14], identifying and addressing compulsive behavioral triggers—when present—through patient education and psychological counseling represents the primary non-pharmacological intervention available for this condition, with potential to limit disease progression.

The differential diagnosis of pachydermodactyly is broad and includes inflammatory arthropathies as well as fibroproliferative, endocrine, and paraneoplastic conditions. Among these are juvenile idiopathic arthritis and rheumatoid arthritis—an overlap with undifferentiated connective tissue disease has also been reported [4]—as well as non-inflammatory entities such as Thiemann disease, true knuckle pads, juvenile fibromatosis, and Garrod’s pads. Overgrowth syndromes (Klippel–Trénaunay, lipomatous macrodystrophy, Proteus syndrome, multiple exostoses syndrome, progressive macrodactyly, digital gigantism, and hyperostotic macrodactyly) should also be considered [29]. Systemic causes, including thyroid acropachy, acromegaly, limited scleroderma, and diffuse systemic sclerosis, as well as rare conditions such as paraneoplastic acropachydermodactyly and hypertrophic pulmonary osteoarthropathy, must also be considered [2,12]. Within the differential diagnosis, knuckle pads are distinguished by their typical location on the dorsal surface of the fingers at the extensor surfaces, usually appearing as well-demarcated lesions. A recent report by Alrubaiaan et al. further highlights the importance of distinguishing PDD from other causes of digital swelling in the gaming-associated behavioral context, where repetitive hand use constitutes a recognized trigger [20]. Thiemann disease, which has its onset in adolescence, presents specific imaging features, including avascular necrosis of the PIP joints, digital deformities, and sclerotic epiphyseal changes with phalangeal shortening. Other rare entities, such as progressive pseudorheumatoid dysplasia or pachydermoperiostosis, may clinically mimic articular involvement but are differentiated by their genetic mechanisms and characteristic osseous changes.

The clinical and pharmacological consequences of misdiagnosis are not merely theoretical. Aljohani reported a 16-year-old male with a three-year history of painless unilateral PIP swelling who was misdiagnosed with JIA and treated with oral methotrexate for one year before PDD was correctly identified—a case that powerfully illustrates the real harm that follows delayed recognition of this condition [2]. The present report, by documenting two further cases of PDD initially suspected as JIA in a pediatric rheumatology center, reinforces the need for systematic inclusion of PDD in the differential diagnosis of painless digital swelling in adolescents, regardless of geographic setting or level of clinical expertise.

From a clinical perspective, the absence of curative treatment necessitates a focus on symptomatic management and psychosocial impact. Although the condition is benign, aesthetic implications may generate anxiety and social isolation, justifying consideration of cosmetic or surgical options in selected cases. Maintaining joint mobility through physiotherapy may prevent long-term complications.

Study Limitations: This report is limited by the small sample size of two cases. Behavioral and psychiatric assessments were not performed using validated instruments. Follow-up duration was limited. Family examination was limited to parents present at admission. Skin biopsy was not performed in either case. The narrative review in Table 2, while representative, is not a systematic review with a formal risk-of-bias assessment.

## 4. Conclusions

PDD is an underrecognized, benign, non-inflammatory fibromatosis that may closely mimic JIA in adolescent patients. The narrative review presented in Table 2 confirms that misdiagnosis as JIA is a consistent finding across multiple clinical settings and geographic regions and that PDD continues to be encountered primarily in rheumatological rather than dermatological practice. The two cases reported here illustrate two clinically distinct subtypes—idiopathic mono-PDD and classic trauma-associated PDD with atypical perilesional hypopigmentation—both encountered within the same pediatric rheumatology service and together represent the first documented cases from Romania. Accurate diagnosis requires systematic exclusion of inflammatory and structural conditions, combining clinical assessment with laboratory and imaging evaluation. MRI, while not a routine requirement, provides valuable soft tissue characterization in diagnostically ambiguous cases and may be justified when atypical clinical features raise concern for inflammatory or infiltrative disease. Clinicians at the rheumatology–dermatology interface should maintain a high index of suspicion for PDD in adolescent males presenting with painless digital swelling to avoid unnecessary immunosuppressive treatment. Family history and behavioral factors should be explored in every case. Multidisciplinary collaboration remains central to optimal patient management.

## Figures and Tables

**Table 1:** Comparative laboratory results for both cases.

Parameter	Case 1	Case 2	Reference Range	Units
Uric Acid	380	290	220–450	µmol/L
ALT (GPT)	17	11	9–24	U/L
AST (GOT)	22	20	14–35	U/L
Total Calcium	2.48	2.44	2.2–2.7	mmol/L
Complement C3	0.97	0.96	0.82–1.85	g/L
Complement C4	0.14	0.14	0.15–0.53	g/L
Creatinine	53	54	58–92	µmol/L
eGFR	114.95	112.39	—	mL/min/1.73 m^2^
Rheumatoid Factor	<20.0	<20.0	<30	IU/mL
Alkaline Phosphatase	317	149	56–167	U/L
Phosphorus	1.41	1.20	1.29–2.26	mmol/L
IgA	1.18	0.88	0.63–4.84	g/L
IgG	12.19	9.92	5.4–18.22	g/L
IgM	1.26	2.05	0.22–2.4	g/L
LDH	223	139	130–250	U/L
LDL Cholesterol	2.00	1.31	0–3.35	mmol/L
Total Lipids	4.57	3.66	5–8	g/L
Magnesium	0.72	0.82	0.7–0.91	mmol/L
CRP	<1.00	<1.00	0–5	mg/L
Triglycerides	0.74	0.34	<1.7	mmol/L
Urea	4.67	3.66	2.6–7.5	mmol/L
D-Dimer HS	74	65	<250	ng/mL
ESR	4	2	2–13	mm/h
ANA	Negative	Negative	Negative	—
Ferritin	30.4	22.8	11.1–171.9	ng/mL
FT4	10.21	11.77	9.98–14.29	pmol/L
TSH	2.10	1.27	0.47–3.41	mIU/L
Vitamin D (25-OH)	21.9	19.1	30–100	ng/mL

**Table 2:** Narrative review of representative published PDD cases (2014–2025) and comparison with the present report.

Author/Year	Age (y)	Sex	Fingers Affected	Trigger	Key Investigations	Misdiagnosis at Referral	Treatment	Clinical Setting
Basex et al., 1973 [3]	14	M	PIP II–IV bilateral	Unknown	Clinical	—	None	Dermatology
Bardazzi et al., 1998 [12]	13–19 (7 cases)	M (all)	PIP II–IV bilateral	Repetitive trauma (4/7)	Clinical, biopsy	JIA suspected	None	Dermatology
Dallos et al., 2014 [6]	Median 14	M > F (4:1)	PIP II–IV	Repetitive trauma, idiopathic	Clinical, XR, US	JIA, RA	None, triamcinolone	Multiple
Gallardo-Villamil et al., 2023 [7]	16	M	PIP II–IV bilateral	None identified	Clinical, XR, biopsy	not reported	Intralesional corticosteroid	Dermatology
Zabotti et al., 2017 [8]	15	M	PIP + MCP bilateral	None identified	US, dermoscopy, biopsy	not reported	None	Rheumatology
Acar et al., 2019 [16]	22, 26	M (both)	PIP II–IV bilateral	Repetitive trauma	US, XR	Inflammatory arthritis	Observation	Rheumatology
Liew & Ting, 2020 [9]	16	M	PIP II–IV bilateral	Kayaking (repetitive)	XR, MRI, biopsy	JIA suspected	Observation + behavior cessation	Rheumatology
Hussain et al., 2021 [17]	39	F	PIP + DIP bilateral	Repetitive hand use	XR, clinical	RA, psoriatic arthritis	Etoricoxib	Rheumatology
Jubber et al., 2021 [18]	17	M	PIP II–IV bilateral	None identified	US, XR, MRI	JIA	Observation	Pediatric Rheumatology
Aljohani, 2022 [2]	14	M	PIP II–IV unilateral	None identified	US, XR	JIA	None	Pediatric Rheumatology
Bocutcu et al., 2022 [5]	14	M	PIP II–IV bilateral	Finger interlacing	Clinical, US	JIA	Observation + counseling	Pediatric Rheumatology
Novais et al., 2022 [19]	15	M	PIP + MCP bilateral	None identified	US, SMI, elastography	Inflammatory arthritis	None	Rheumatology
Alrubaiaan et al., 2025 [20]	14	M	PIP II–IV bilateral	Gaming (5 h/day)	Clinical, MRI	not reported	None	Dermatology
Rukavina et al., 2025 (7 cases) [14]	12–16 (median 14)	M 6/7, F 1/7	PIP II–IV (all)	Repetitive trauma (4/7)	US, XR, MRI (selected)	JIA (all)	None or observation	Pediatric Rheumatology
Almeida et al., 2025 [21]	14	F	PIP II–III bilateral	Piano lessons	Clinical, XR	not reported	Triamcinolone infiltration	Rehab Medicine
Present study—Case 1 *	13 y 5 m	M	PIP II–IV (predominantly unilateral)	None (idiopathic)	Clinical, labs, US, XR	JIA suspected	None	Pediatric Rheumatology (Romania)
Present study—Case 2 *	16	M	PIP II–IV bilateral + PIP V (R)	Repetitive finger-twisting	Clinical, labs, US, XR, MRI	JIA suspected	Behavioral counseling	Pediatric Rheumatology (Romania)

* Present study cases. F = female; M = male; PIP = proximal interphalangeal; MCP = metacarpophalangeal; DIP = distal interphalangeal; US = ultrasonography; XR = radiography; MRI = magnetic resonance imaging; SMI = superb microvascular imaging; JIA = juvenile idiopathic arthritis; RA = rheumatoid arthritis.

## Data Availability

The data are not publicly available due to privacy considerations.
